# Dysapolipoproteinaemia Influences the Relationship Between Very Low-Density Lipoprotein Cholesterol and Intra-Pancreatic Fat Deposition in Humans

**DOI:** 10.3390/nu17233718

**Published:** 2025-11-27

**Authors:** Yutong Liu, Loren Skudder-Hill, Juyeon Ko, Xiatiguli Shamaitijiang, Ivana R. Sequeira-Bisson, Maxim S. Petrov

**Affiliations:** 1 School of Medicine, University of Auckland, Auckland 1023, New Zealand; 2 Yong Loo Lin School of Medicine, National University of Singapore, Singapore 119077, Singapore; 3 College of Medicine, Yonsei University, Seoul 03722, Republic of Korea; 4 Human Nutrition Unit, School of Biological Sciences, University of Auckland, Auckland 1010, New Zealand

**Keywords:** intra-pancreatic fat deposition, very low-density lipoprotein cholesterol, apolipoprotein B, apolipoprotein C-II, apolipoprotein C-III

## Abstract

**Background:** Apolipoprotein B (apo B), apolipoprotein C-II (apo C-II), and apolipoprotein C-III (apo C-III) play important roles in very low-density lipoprotein (VLDL) metabolism. Whether they influence the relationship between intra-pancreatic fat deposition (IPFD) and VLDL is unknown. The aim was to investigate whether the association between VLDL cholesterol (VLDL-C) and IPFD varies between individuals with and without dysapolipoproteinaemia involving apo B, apo C-II, and apo C-III. **Methods:** Abdominal magnetic resonance imaging at 3T was performed to quantify IPFD. VLDL-C was measured using the Quantimetrix Lipoprint^®^ system, whereas apo B, apo C-II, and apo C-III levels were analysed using the MILLIPLEX^®^ (xMAP) assay. Dysapolipoproteinemia was defined as apolipoprotein levels above the upper quartile of the overall cohort. Univariable and multivariable linear regression analyses were performed, adjusting for age, sex, ethnicity, waist-to-hip ratio, high-density lipoprotein cholesterol, and insulin resistance. **Results:** A total of 32 individuals had dysapolipoproteinaemia, whereas 96 had normoapolipoproteinaemia. Among those with dysapolipoproteinaemia involving apo B, apo C-II, and apo C-III, VLDL-C levels were significantly and positively associated with IPFD. In the fully adjusted model, each unit increase in VLDL-C corresponded to a 0.82% (*p* = 0.011), 1.05% (*p* = 0.003), and 1.00% (*p* = 0.005) increase in IPFD, respectively. No significant association between VLDL-C and IPFD was observed in individuals with normoapolipoproteinaemia. **Conclusions:** Altered apolipoprotein profiles influence the association between VLDL-C and IPFD.

## 1. Introduction

Excessive intra-pancreatic fat deposition (IPFD) is the most common pancreatic pathology, affecting approximately one-fifth of the general population [1,2]. Evidence also suggests a sex difference, with men typically exhibiting significantly higher IPFD than women [3,4]. Far from being merely a consequence of general adiposity, excessive IPFD is suggested to underlie the development of diseases of the exocrine pancreas, conferring an increased risk of pancreatitis and pancreatic cancer [5,6]. Beyond its involvement in diseases of the exocrine pancreas, excessive IPFD has notable metabolic implications, with evidence from both cross-sectional and longitudinal studies linking it to significantly elevated risks of insulin resistance [7], diabetes mellitus [8], and metabolic syndrome [9]. Emerging evidence also implicates excessive IPFD as an underappreciated risk factor for cardiovascular diseases (CVD). A 2025 meta-analysis of human studies suggested that fat in the pancreas is associated with a wide range of macrovascular and microvascular manifestations, including subclinical atherosclerosis, increased carotid intima thickness, vascular stiffness, and vessel calcification [10]. One mechanistic explanation for this link between excessive IPFD and CVD is the involvement of dyslipidaemia. Excessive IPFD has been shown to be significantly associated with dyslipidaemias, including low high-density lipoprotein cholesterol [11,12] and elevated triglyceride-rich lipoproteins in humans [13,14].

Triglyceride-rich lipoproteins, particularly very low-density lipoprotein (VLDL), have emerged as independent predictors of residual CVD risk, contributing to in-stent restenosis [15], increased carotid intima-media thickness [16], vascular stiffness [17], and coronary artery calcification, even independently of plasma triglyceride levels [18,19]. Although increased VLDL production driven by hepatic fat deposition was previously hypothesised as a potential driver of excessive IPFD [20,21], a 2025 study reported no significant association between VLDL-cholesterol (VLDL-C) and IPFD in individuals from the general population [14]. The reason for this discrepancy remains unclear. Apolipoproteins, particularly apolipoprotein B (apo B), apolipoprotein C-II (apo C-II), and apolipoprotein C-III (apo C-III), may contribute to this relationship [22]. Apo B, the structural protein of VLDL, has been proposed as a proxy marker of VLDL particle number due to its 1:1 stoichiometry with VLDL [23]. Additionally, both apo C-II and apo C-III play key roles in VLDL metabolism [24,25,26]. Apo C-II acts as a cofactor for lipoprotein lipase (LPL), facilitating the clearance of VLDL [27], whereas apo C-III is thought to inhibit the LPL-mediated hydrolysis of VLDL triglycerides (thereby delaying their clearance) [28]. Furthermore, a 2025 study reported a significant association between apo C-III and IPFD, identifying apo-C-III as a contributor to the relationship between IPFD and triglyceride-rich lipoproteins [29]. Given that apolipoproteins—particularly apo B, apo C-II, and apo C-III—are intimately involved in both VLDL metabolism and IPFD, we hypothesised that the relationship between IPFD and VLDL may vary according to the levels of these apolipoproteins.

The primary aim of this study was to investigate the association of VLDL-C and IPFD in individuals with and without dysapolipoproteinaemia involving apo B, apo C-II, and apo C-III. The secondary aim was to examine the associations between the studied apolipoproteins and VLDL-C within their respective subgroups.

## 2. Methods

### 2.1. Study Population

The design of the present study was cross-sectional. Inclusion criteria comprised adults (both men and women) residing in Auckland, New Zealand, who underwent magnetic resonance imaging (MRI) of the pancreas for research purposes and provided a blood sample following an overnight fast of 8–10 h. Participants were excluded if they had pancreatic cancer, chronic pancreatitis, congenital pancreatic abnormalities, pancreatic lipoma, pancreatic trauma, or cystic fibrosis. Individuals with a history of pancreatic surgery or endoscopic procedures were also excluded. Further exclusion criteria were type 1 diabetes, gestational diabetes, liver disease, malignancy, autoimmune disorders, cognitive impairment, and pregnancy or breastfeeding. MRI-related contraindications, such as the presence of metallic implants, pacemakers, or other electronic devices, or severe chronic obstructive pulmonary disease limiting breath-holding, also resulted in exclusion. Written informed consent was obtained from all participants prior to enrolment.

### 2.2. Quantification of Intra-Pancreatic Fat Deposition

All participants underwent an abdominal MRI scan using the same 3.0 Tesla MAGNETOM scanner (Siemens Healthineers, Erlangen, Germany) at the Centre for Advanced MRI (University of Auckland), using the same standardised imaging protocol that has been previously described in detail [30,31]. IPFD was quantified using a modified ‘MR-opsy’ technique [32]. In brief, two slices of the pancreas, each 5 mm in thickness, were selected using the MicroDicom software version 2023.2 (MicroDicom, Sofia, Bulgaria). Within each slice, three regions of interest were placed over the head, body, and tail of the pancreas using the ImageJ software version 1.54 (National Institutes of Health, Bethesda, Rockville, MD, USA). A threshold range of 1–20% was applied to exclude non-parenchymal tissues, including peripancreatic fat and vascular tissues. IPFD quantification was then carried out by two independent raters, each assessing the two candidate slices, with the final IPFD value calculated as the average of their measurements.

### 2.3. Quantification of Very Low-Density Lipoprotein Cholesterol

Blood samples for VLDL-C measurements were centrifuged at 4000× *g* for 5.5 min at 4 °C. Serum was separated into aliquots and stored at −80 °C until batch analysis. VLDL-C was measured using the Lipoprint^®^ lipoprotein subfractions testing system (Quantimetrix Corp., Redondo Beach, CA, USA) in accordance with the manufacturer’s instructions, with the detailed protocol published elsewhere [14]. In brief, the proportion of VLDL was expressed as the percentage of the area under the curve (%), representing the area of the VLDL band relative to the total cholesterol-bound area of all detected lipoprotein bands in the Lipoprint profile. The concentration of VLDL-C was then calculated by multiplying the relative area of VLDL (%) by the total cholesterol concentration of the sample in mg/dL.

### 2.4. Other Measurements

Following an overnight fast of at least 8 h, venous blood samples were drawn into EDTA and lithium heparin tubes. Levels of apo B, apo C-II, and apo C-III were determined using the MILLIPLEX^®^ MAP Human Apolipoprotein Magnetic Bead Panel (Cat # HAP0-8062; Millipore, Billerica, MA, USA). The intra- and inter-assay coefficients of variation were <10% and <20%, respectively. Dysapolipoproteinaemia was defined as the corresponding apolipoprotein level exceeding the upper quartile (75th percentile) of the overall cohort, whereas normoapolipoproteinaemia was defined as having the corresponding apolipoprotein level at or below the upper quartile of the overall cohort.

Fresh, never-frozen blood samples were sent to LabPLUS (Auckland City Hospital, New Zealand), an accredited tertiary medical laboratory, for measurements of total cholesterol (mg/dL), LDL cholesterol (mg/dL), HDL cholesterol (mg/dL), triglycerides (mg/dL), fasting insulin (mU/L), fasting plasma glucose (mmol/L), and haemoglobin A1c (HbA1c, mmol/mol). Insulin resistance was estimated using the Homeostasis Model Assessment of Insulin Resistance (HOMA-IR) index, calculated as fasting serum insulin (μU/mL) multiplied by fasting plasma glucose (mmol/L) and divided by 22.5 [33].

Demographic information, including age, ethnicity, and sex, was collected from all participants using a standardised form. Anthropometric measurements, including body weight (kg), height (cm), and waist and hip circumferences (cm), were taken in duplicate following a consistent protocol [34]. The average of the two measurements was used for analysis. Body mass index was calculated by dividing weight (kg) by height (m) squared. The waist-to-hip ratio was calculated by dividing the waist circumference (cm) by the hip circumference (cm).

### 2.5. Statistical Analysis

Statistical analyses were performed using IBM SPSS version 29.0.1.0 for Macintosh (IBM Corp., Armonk, New York, NY, USA). Across all variables, less than 5% of data were missing completely at random, and these missing values were handled using both the last-observation-carried-forward method and the multiple imputation method. The variables included in the imputation process were age, sex, ethnicity, body weight, height, IPFD, waist circumference, hip circumference, fasting plasma glucose, HbA1c, fasting insulin, triglycerides, total cholesterol, LDL cholesterol, and HDL cholesterol. The distribution of continuous variables was assessed using the Shapiro–Wilk test. Variables with skewed distributions, namely IPFD, age, waist-to-hip ratio, HDL cholesterol, apo B, apo C-II, and apo C-III, were log-transformed prior to analyses. Continuous variables were expressed as the median (interquartile range), and categorical variables were presented as frequencies and percentages. Comparisons of baseline characteristics between the dysapolipoproteinaemia and normoapolipoproteinaemia subgroups for each apolipoprotein were performed using Student’s *t*-tests, with results reported as the median (interquartile range). Further, univariable and multivariable linear regression analyses were carried out to investigate the associations between continuous variables of interest. Three statistical models were built: the unadjusted model (Model 1), the model adjusted for age, sex, and ethnicity (Model 2), and the model adjusted for age, sex, ethnicity, waist-to-hip ratio, HOMA-IR, and HDL cholesterol (Model 3). Regression outcomes were reported as β coefficients with 95% confidence intervals and the corresponding *p*-values. Statistical significance was defined as a two-tailed *p*-value < 0.05.

## 3. Results

### 3.1. Overall Characteristics

A total of 137 individuals met the eligibility criteria. Seven individuals were excluded due to missing total cholesterol data, and two were excluded due to MRI artefacts, resulting in a cohort of 128 individuals for analysis. Of these, 76 (59.4%) were men and 52 (40.6%) were women. The median (interquartile range) levels of VLDL-C were 40.65 (34.30, 53.12) mg/dL. The median (interquartile range) levels of the studied apolipoproteins were as follows: apo B, 71.16 mg/dL (24.85–2160.30 mg/dL); apo C-II, 5.05 mg/dL (1.99–56.83 mg/dL); and apo C-III, 15.10 mg/dL (7.69–79.28 mg/dL). Based on apolipoprotein levels, 32 individuals were categorised as having dysapolipoproteinaemia and 96 as having normoapolipoproteinaemia. Notably, 93.8% (n = 30) of individuals in the dysapolipoproteinaemia subgroup overlapped across the apo B, apo C-II, and apo C-III groups. Additional characteristics of the study cohort are presented in Table 1.

### 3.2. Comparison of Apolipoproteinaemia Subgroups

There was a significant difference in sex distribution between the dysapolipoproteinaemia and normoapolipoproteinaemia subgroups of apo B, with a higher proportion of women in the dysapolipoproteinaemia subgroup compared with the normoapolipoproteinaemia subgroup (56.3% vs. 35.4%, *p* = 0.038). No significant differences were observed in other demographic characteristics across the dysapolipoproteinaemia and normoapolipoproteinaemia subgroups of apo B, apo C-II, or apo C-III (Table 2).

### 3.3. Association Between Intra-Pancreatic Fat Deposition and Very Low-Density Lipoprotein Cholesterol Across Apolipoproteinaemia Subgroups

**Figure 1:**
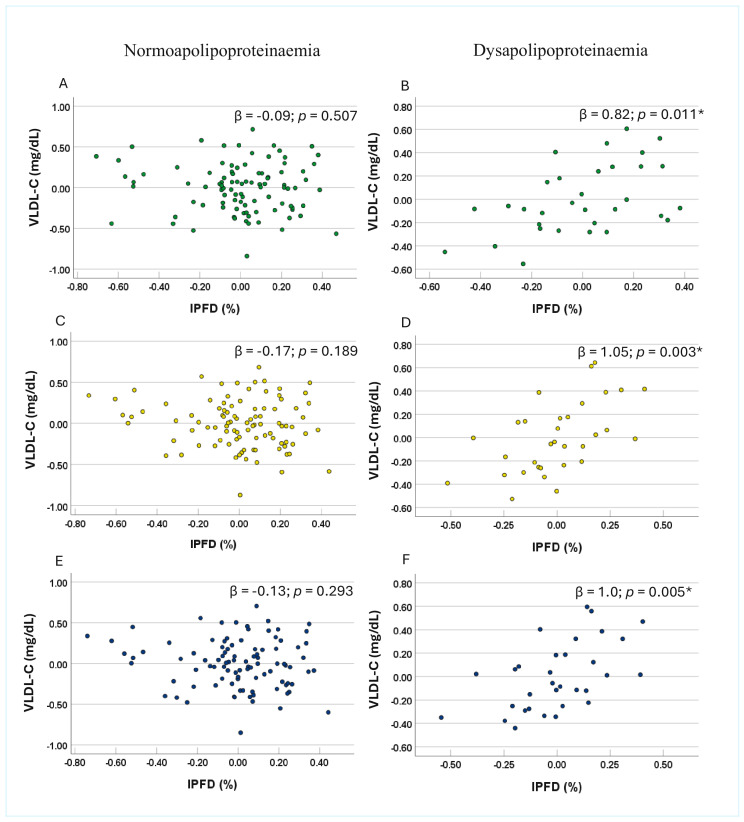
Associations between very low-density lipoprotein cholesterol and intra-pancreatic fat deposition in the subgroups involving apolipoprotein B (panel (**A**) and panel (**B**)), apolipoprotein C-II (panel (**C**) and panel (**D**)), and apolipoprotein C-III (panel (**E**) and panel (**F**)) dysapolipoproteinaemia and normoapolipoproteinaemia. Footnotes: Data are presented as partial residual plots. *p*-values and β-coefficients were derived from multivariable analysis after adjusting for age, sex, ethnicity, waist-to-hip ratio, homeostatic model assessment of insulin resistance, and high-density lipoprotein cholesterol in the dysapolipoproteinaemia (n = 32) and normoapolipoproteinaemia (n = 96) subgroups. Data for intra-pancreatic fat deposition, age, waist-to-hip ratio, high-density lipoprotein cholesterol, apo B, apo C-II, and apo C-III were log-transformed. * Indicates significant associations (*p* < 0.05). Abbreviations: IPFD—intra-pancreatic fat deposition; VLDL-C —very low-density lipoprotein cholesterol.

No significant association between IPFD and VLDL-C was observed in either the normoapolipoproteinaemia or dysapolipoproteinaemia subgroups of apo B, apo C-II, or apo C-III in Model 1 (Table 3). However, in Model 2, IPFD was significantly and positively associated with VLDL-C in the dysapolipoproteinaemia subgroups of apo B, apo C-II, and apo C-III, whereas no significant associations were observed in the corresponding normoapolipoproteinaemia subgroups (Table 3). Similarly, in Model 3, significant positive associations between IPFD and VLDL-C were observed in the dysapolipoproteinaemia subgroups, with each unit increase in IPFD corresponding to a 0.82-unit (*p* = 0.011), 1.05-unit (*p* = 0.003), and 1.00-unit (*p* = 0.005) increase in VLDL-C for apo B, apo C-II, and apo C-III, respectively (Figure 1B,D,E). In contrast, no significant associations were observed in any of the normoapolipoproteinaemia subgroups across unadjusted or adjusted models (Figure 1A,C,E).

### 3.4. Association Between Apolipoproteins and Very Low-Density Lipoprotein Cholesterol Across Apolipoproteinaemia Subgroups

No significant association of apo B, apo C-II, or apo C-III with VLDL-C was observed in any of the subgroups in the unadjusted model, after adjusting for age, sex, and ethnicity, or in the most adjusted model (Table 4).

## 4. Discussion

This study is the first to have investigated the relationship between VLDL-C and IPFD (assessed using gold-standard 3.0-Tesla MRI) in individuals with and without dysapolipoproteinaemia involving apo B, apo C-II, and apo C-III. The primary finding is that IPFD was significantly and positively associated with VLDL-C in the dysapolipoproteinaemia subgroups, consistently across all studied apolipoproteins. Notably, these associations remained robust after adjusting for demographic characteristics (age, sex, and ethnicity), anthropometric measurements (waist-to-hip ratio), markers of insulin resistance (HOMA-IR), and markers of dyslipidaemia (HDL cholesterol)—all factors previously shown to influence both IPFD and VLDL [35,36,37,38,39]. Furthermore, there was approximately 95% overlap among the dysapolipoproteinaemia subgroups for three apolipoproteins studied, indicating that a substantial proportion of individuals had dysapolipoproteinaemic profiles across all markers, potentially placing them at higher risk for CVD.

Although the relationship between fat in the pancreas and VLDL has been previously investigated, earlier theories proposed that increased VLDL-triglyceride production from hepatic de novo lipogenesis contributes to excessive IPFD [1,20]. However, a 2025 cross-sectional study conducted by our research group did not support this hypothesis, as no significant association between IPFD and VLDL was observed [14]. One of the key findings of the present study is the identification of a distinct subgroup—individuals with apo B dysapolipoproteinaemia—that exhibits a significantly positive association between IPFD and VLDL-C. As a principal structural component of apo-B-containing lipoproteins, including VLDL, apo B has long been regarded as a proxy for the number of VLDL particles, since each VLDL particle contains a single apo B molecule [40]. Epidemiological evidence increasingly supports apo B as a superior predictor of CVD risks compared with traditional markers of dyslipidaemia—such as total cholesterol or LDL cholesterol [41,42]. This reinforces the notion that CVD risk is primarily driven by the number of atherogenic lipoprotein particles, as indexed by apo B, rather than by total circulating cholesterol or triglycerides [42]. Moreover, VLDL bound to apo B (VLDL-apo B) has been shown to reflect the burden of atherogenic particles capable of arterial penetration [22]. Importantly, the present study provided deeper mechanistic insights into the pathogenesis of excessive IPFD and its link with triglyceride-rich lipoprotein, as the association between IPFD and atherogenic VLDL-C was observed only in the subgroup at high CVD risks—those with apo B dysapolipoproteinaemia. 

Insulin resistance, which is known to increase apo B secretion [43,44] and reduce its degradation [45], may explain the observed association. It has been hypothesised that hepatic insulin resistance exacerbates de novo lipogenesis, thereby increasing VLDL-triglyceride production. This excess hepatic VLDL-triglyceride is subsequently exported to the pancreas, contributing to IPFD. Excessive IPFD, in turn, promotes pancreatic β-cell dysfunction, initially compensated by increased insulin secretion until β-cells become overwhelmed, thereby reinforcing a self-perpetuating cycle [38]. It has been proposed that increased hepatic production of VLDL in insulin resistance may result from altered function of microsomal triglyceride transfer protein, which plays a crucial role in transferring neutral lipids to nascent apo B and represents a rate-limiting step in hepatic VLDL production [46,47]. Moreover, a human study has demonstrated that insulin resistance is an independent predictor of VLDL-apo B hepatic secretion [48]. Additionally, emerging evidence supports a strong association between IPFD and insulin resistance [37,49]. Taken together, the positive association between IPFD and VLDL observed in individuals with apo B dysapolipoproteinaemia is, at least in part, attributable to insulin resistance related to excessive IPFD. 

A significant positive association between IPFD and VLDL-C was also found in individuals with apo C-II and apo C-III dysapolipoproteinaemia. Similar to apo B, both apo C-II and apo C-III are exchangeable apolipoproteins found on VLDL particles, where they play key roles in VLDL metabolism [50]. Recent evidence indicates that, at high levels, apo C-II downregulates LPL activity rather than promoting it [51]. Overexpression of the human *APOC2* gene has been associated with marked hypertriglyceridaemia, primarily due to the accumulation of large, TG-rich VLDL particles, suggesting a lipolytic defect [52]. It has been hypothesised that this lipolytic defect may result from impaired access of LPL to triglyceride-rich lipoproteins [52]. Furthermore, a recent longitudinal study of more than 3000 individuals, followed for nearly 10 years, demonstrated an inverse J-shape relationship between apo C-II and CVD mortality, with the lowest CVD risk observed in the middle quintile and increased risk at both extremes [53]. A corresponding inverse trend has been reported with regard to LPL activity [53]. Both in vivo and in vitro studies have shown that apo C-III promotes VLDL assembly and secretion [54,55,56,57,58]. Apo C-III also inhibits VLDL lipolysis by weakening the binding of VLDL to the capillary endothelium, which is the primary site of LPL activity [59]. Moreover, apo C-III has consistently been identified as an independent predictor of CVD [22,60]. Mendelian randomisation analyses further support a causal relationship, showing that individuals lacking functional apo C-III have approximately 40% lower triglyceride levels and reduced CVD risk compared with their healthy counterparts [61,62]. Collectively, the present study provides novel evidence of a significant positive association between IPFD and VLDL in individuals at high CVD risk, characterised by dysapolipoproteinaemia of apo C-II and C-III.

Several limitations of this study should be acknowledged. First, the cross-sectional design precludes any inference regarding the temporal or causal nature of the observed associations; longitudinal studies and randomised controlled trials are warranted to address this. Second, while we demonstrated statistically significant associations between VLDL-C and IPFD in individuals with dysapolipoproteinaemia of apo B, apo C-II, and C-III, the small sample size (n = 32) of this subgroup may limit statistical power and increase the risk of type II error. Larger studies are warranted to validate these associations. Third, only total apo B was measured. Apo B exists in two isoforms, apo B-48 and apo B-100 [63]. Given that apo B-48 is primarily associated with chylomicrons, its concentration is expected to be minimal in fasting blood samples, which were used in the present study. Fourth, hepatic fat deposition–which was not accounted for in the present study–has been shown to influence the metabolism of triglyceride-rich lipoproteins. Future studies may consider investigating how hepatic fat deposition affects the studied associations. Fifth, dysapolipoproteinaemia status was defined using cohort-specific upper quartile cutoffs, which limit comparability with external populations. Sixth, VLDL-C concentration was derived from total cholesterol concentration (mg/dL) and the percentage VLDL (%VLDL). Consequently, it does not reflect VLDL particle number or size, both of which may have independent CVD implications [64]. Previous studies have demonstrated that distinct VLDL subfractions convey varying levels of cardiovascular risk [65]. Future studies should consider measuring VLDL particle size or quantifying VLDL particle number using methods such as ultracentrifugation.

## 5. Conclusions

VLDL-C was positively associated with IPFD only in individuals with dysapolipoproteinaemia of apo B, apo C-II, or apo C-III, whereas no significant association was observed in normoapolipoproteinaemic individuals. These findings suggest that alterations in apolipoprotein levels may influence the relationship between VLDL-C and IPFD. Accordingly, assessing apolipoprotein profiles may help identify individuals in whom elevated VLDL-C is linked with excessive IPFD, providing valuable insights into their CVD risk.

## Figures and Tables

**Table 1:** Participant characteristics.

Characteristic	Overall (n = 128)
Age (years)	57.60 (43.56, 67.29)
Ethnicity (%)	
− NZ Europeans	n = 50 (39.06%)
− Māori	n = 21 (16.41%)
− Pacific Islander	n = 3 (2.34%)
− Asian	n = 27 (21.09%)
− Other	n = 27 (21.09%)
Waist-to-hip ratio	0.96 (0.88, 1.01)
Body mass index (kg/m^2^)	27.11 (23.57, 31.12)
HbA1c (mmol/mol)	36.97 (35.16, 40.04)
HOMA-IR	3.37 (1.45, 4.06)
Total cholesterol (mg/dL)	177.68 (151.41, 208.51)
LDL-C (mg/dL)	100.54 (73.47, 126.64)
HDL-C (mg/dL)	50.30 (41.83, 61.90)
Triglycerides (mg/dL)	111.05 (79.84, 169.02)

Footnote: Continuous variables are reported as the median and interquartile range, whereas categorical variables are presented as the frequency and percentage. Abbreviations: HbA1c—haemoglobin A1c; HDL-C—high-density lipoprotein cholesterol; HOMA-IR—homeostatic model assessment for insulin resistance; LDL-C—low-density lipoprotein cholesterol.

**Table 2:** Comparison of baseline characteristics between the study subgroups.

	Apolipoprotein B			Apolipoprotein C-II			Apolipoprotein C-III		
Characteristic	Normo- Apolipoproteinaemia (n = 96)	Dys- Apolipoproteinaemia (n = 32)	*p*	Normo- Apolipoproteinaemia (n = 96)	Dys- Apolipoproteinaemia (n = 32)	*p*	Normo- Apolipoproteinaemia (n = 96)	Dys- Apolipoproteinaemia (n = 32)	*p*
Age (years)	58.26 (41.16, 65.86)	55.98 (40.45, 68.71)	0.848	57.40 (43.49, 64.72)	55.98 (37.62, 70.46)	0.781	57.40 (42.41, 64.72)	57.40 (38.67, 69.23)	0.978
Sex (%)			**0.038**			0.098			0.216
− Men	n = 62 (64.58%)	n = 14 (43.75%)		n = 61 (63.54%)	n = 15 (46.88%)		n = 60 (62.50%)	n = 16 (50%)	
− Women	n = 34 (35.42%)	n = 18 (56.25%)		n = 35 (36.46%)	n = 17 (53.13%)		n = 36 (37.50%)	n = 16 (50%)	
Ethnicity (%)			0.999			0.621			0.621
− NZ Europeans	n = 37 (38.54%)	n = 13 (40.63%)		n = 37 (38.54%)	n = 13 (40.63%)		n = 37 (38.54%)	n = 13 (40.63%)	
− Māori	n = 17 (17.71%)	n = 4 (12.50%)		n = 18 (18.75%)	n = 3 (9.38%)		n = 18 (18.75%)	n = 3 (9.38%)	
− Pacific Islander	n = 2 (2.08%)	n = 1 (3.13%)		n = 2 (2.08%)	n = 1 (3.13%)		n = 2 (2.08%)	n = 1 (3.13%)	
− Asian	n = 19 (19.79%)	n = 8 (25.0%)		n = 20 (20.83%)	n = 7 (21.88%)		n = 20 (20.83%)	n = 7 (21.88%)	
− Other	n = 21 (21.88%)	n = 6 (18.75%)		n = 19 (19.79%)	n = 8 (25.0%)		n = 19 (19.79%)	n = 8 (25.0%)	
Waist-to-hip ratio	0.96 (0.89, 1.01)	0.94 (0.87, 0.99)	0.132	0.96 (0.89, 1.01)	0.95 (0.87, 1.01)	0.625	0.96 (0.89, 1.01)	0.95 (0.87, 1.01)	0.546
Body mass index (kg/m^2^)	27.11 (24.05, 31.11)	25.03 (21.76, 31.11)	0.328	27.11 (24.05, 31.11)	25.28 (22.14, 31.11)	0.242	27.11 (23.87, 30.88)	26.31 (23.34, 31.19)	0.430
HOMA-IR	2.45 (1.56, 4.23)	2.13 (1.17, 3.95)	0.159	2.46 (1.59, 4.96)	1.99 (1.07, 3.48)	0.066	2.45 (1.56, 4.96)	2.17 (1.16, 3.50)	0.085
HbA1c (mmol/mol)	36.97 (35.16, 40.65)	36.97 (34.38, 39.55)	0.399	36.97 (35.16, 40.85)	36.42 (34.12, 38.09)	0.077	36.97 (35.16, 40.85)	36.05 (33.36, 38.67)	0.075
Total cholesterol (mg/dL)	179.47 (151.41, 211.66)	175.91 (140.47, 205.41)	0.472	181.27 (151.41, 215.94)	175.91 (140.47, 205.41)	0.106	181.27 (151.41, 215.94)	172.43 (140.47, 195.39)	0.224
LDL-C (mg/dL)	100.54 (73.47, 122.78)	102.48 (74.44, 130.51)	0.736	104.41 (77.34, 126.64)	92.81 (66.71, 125.68)	0.282	102.48 (77.34, 126.64)	96.68 (66.71, 125.68)	0.403
HDL-C (mg/dL)	50.40 (39.06, 57.97)	50.40 (38.86, 68.55)	0.303	50.40 (38.86, 57.97)	48.42 (39.75, 71.52)	0.761	50.40 (38.86, 57.97)	50.40 (39.75, 73.33)	0.454

Footnotes: Results are from Student’s *t*-test comparing the normoapolipoproteinaemia subgroup versus the dysapolipoproteinaemia subgroup. Data are presented as median (interquartile range) or frequency (percentage) and *p* values. Statistically significant values (*p* < 0.05) are shown in bold. Abbreviations: HbA1c—haemoglobin A1c; HDL-C—high-density lipoprotein cholesterol; HOMA-IR—homeostatic model assessment for insulin resistance; LDL-C—low-density lipoprotein cholesterol.

**Table 3:** Association between very low-density lipoprotein cholesterol and intrapancreatic fat deposition in the normoapolipoproteinaemia versus dysapolipoproteinaemia subgroups.

Apolipoprotein	Subgroup	Model 1		Model 2		Model 3	
		β (95% CI)	*p*	β (95% CI)	*p*	β (95% CI)	*p*
Apo B	Normoapolipoproteinaemia	−0.09 (−0.26, 0.14)	0.465	−0.04 (−0.27, 0.19)	0.732	−0.09 (−0.34, 0.17)	0.507
	Dysapolipoproteinaemia	0.29 (−0.05, 0.64)	0.104	**0.50 (0.06, 0.93)**	**0.034**	**0.82 (0.23, 1.41)**	**0.011**
Apo C-II	Normoapolipoproteinaemia	−0.12 (−0.33, 0.08)	0.231	−0.12 (−0.33, 0.10)	0.291	−0.17 (−0.41, 0.08)	0.189
	Dysapolipoproteinaemia	0.26 (−0.39, 0.91)	0.149	**0.59 (0.04, 1.13)**	**0.025**	**1.05 (0.45, 1.65)**	**0.003**
Apo C-III	Normoapolipoproteinaemia	−0.11 (−0.32, 0.11)	0.309	−0.11 (−0.35, 0.14)	0.345	−0.13 (−0.43, 0.17)	0.293
	Dysapolipoproteinaemia	0.26 (−0.08, 0.61)	0.148	**0.70 (−0.13, 1.52)**	**0.007**	**1.00 (−0.11, 2.10)**	**0.005**

Footnotes: Results are from the univariate and multivariate linear regression analyses of very low-density lipoprotein and intra-pancreatic fat deposition in 128 participants. Data are presented as β coefficients (95% CI) and *p* values. Statistically significant values (*p* < 0.05) are shown in bold. Model 1 was unadjusted; Model 2 was adjusted for age, sex, and ethnicity; Model 3 was adjusted for age, sex, ethnicity, waist-to-hip ratio, homeostatic model assessment of insulin resistance, and high-density lipoprotein cholesterol. Values for intra-pancreatic fat deposition, age, waist-to-hip ratio, high-density lipoprotein cholesterol, apo B, apo C-II, and apo C-III were log-transformed prior to analysis. Abbreviations: apo B—apolipoprotein B; apo C-II—apolipoprotein C-II; apo C-III—apolipoprotein C-III; CI—confidence interval.

**Table 4:** Associations between apolipoproteins and very low-density lipoprotein cholesterol in the normoapolipoproteinaemia versus dysapolipoproteinaemia subgroups.

Apolipoprotein	Subgroup	Model 1		Model 2		Model 3	
		β (95% CI)	*p*	β (95% CI)	*p*	β (95% CI)	*p*
Apo B	Normoapolipoproteinaemia	−0.16 (−0.36, 0.04)	0.121	−0.19 (−0.40, 0.02)	0.078	−0.18 (−0.40, 0.03)	0.098
	Dysapolipoproteinaemia	−0.30 (−0.64, 0.05)	0.100	−0.31 (−0.73, 0.12)	0.167	−0.34 (−0.83, 0.16)	0.197
Apo C-II	Normoapolipoproteinaemia	0.04 (−0.17, 0.24)	0.717	0.03 (−0.18, 0.24)	0.786	0.04 (−0.13, 0.20)	0.732
	Dysapolipoproteinaemia	−0.10 (−0.45, 0.26)	0.587	−0.07 (−0.46, 0.31)	0.720	−0.23 (−0.68, 0.22)	0.327
Apo C-III	Normoapolipoproteinaemia	0.01 (−0.19, 0.22)	0.892	0.01 (−0.19, 0.20)	0.962	0.02 (−0.20, 0.24)	0.850
	Dysapolipoproteinaemia	−0.11 (−0.46, 0.25)	0.550	−0.09 (−0.46, 0.29)	0.648	−0.14 (−0.54, 0.26)	0.508

Footnotes: Results are from the univariate and multivariate linear regression analyses of very low-density lipoprotein cholesterol and the three studied apolipoproteins in their respective subgroups of normoapolipoproteinaemia versus dysapolipoproteinaemia. Data are presented as β coefficients (95% CI) and *p* values. Model 1 was unadjusted; Model 2 was adjusted for age, sex, and ethnicity; Model 3 was adjusted for age, sex, ethnicity, waist-to-hip ratio, homeostatic model assessment of insulin resistance, and high-density lipoprotein cholesterol. Values for intra-pancreatic fat deposition, age, waist-to-hip ratio, high-density lipoprotein cholesterol, apo B, apo C-II, and apo C-III were log-transformed prior to analysis. Abbreviations: apo B—apolipoprotein B; apo C-II—apolipoprotein C-II; apo C-III—apolipoprotein C-III; CI—confidence interval.

## Data Availability

Some or all datasets generated during and/or analysed during the current study are not publicly available due to privacy restrictions but are available from the corresponding author upon reasonable request.
